# Comparison of the oxygenation index and the oxygen saturation index as clinical indicators for neonatal ECMO

**DOI:** 10.3389/fped.2025.1586985

**Published:** 2025-06-24

**Authors:** John Slaughter, Jeremy Sites, Hubert Ballard, John Bauer, Aric Schadler, Nicholas Severyn

**Affiliations:** ^1^ University of Kentucky College of Medicine, Lexington, KY, United States; ^2^ Department of Pediatrics, University of Kentucky College of Medicine, Kentucky Children’s Hospital, Lexington, KY, United States; ^3^ University of Kentucky, Lexington, KY, United States

**Keywords:** neonatal ECMO, oxygenation index, oxygen saturation index, persistent pulmonary hypertension, neonatal hypoxemic respiratory failure

## Abstract

**Introduction:**

Neonatal hypoxic respiratory failure is commonly assessed with the oxygenation index (OI) to determine severity and guide ECMO initiation. Calculation of the OI requires arterial blood sampling which can be difficult to obtain. A non-invasive alternative, the oxygen saturation index (OSI), has shown promise, but its utility in ECMO determination is not well-described. We aimed to evaluate the correlation between the OI and OSI in neonates requiring ECMO.

**Methods:**

We pursued a retrospective chart review of 64 neonatal ECMO patients at Kentucky Children's Hospital (2012–2022) and analyzed OI and OSI values in the 12 h preceding ECMO initiation.

**Results:**

A moderate correlation was observed between the OI and OSI. An OSI >17.41 predicted ECMO initiation, and OI can be estimated with the equation: OI = 1.978(OSI)—6.743.

**Conclusion:**

These findings suggest OSI may be a useful adjunct to OI for assessing neonatal respiratory failure and could be beneficial when arterial sampling is impractical.

## Introduction/background

**Figure 1:**
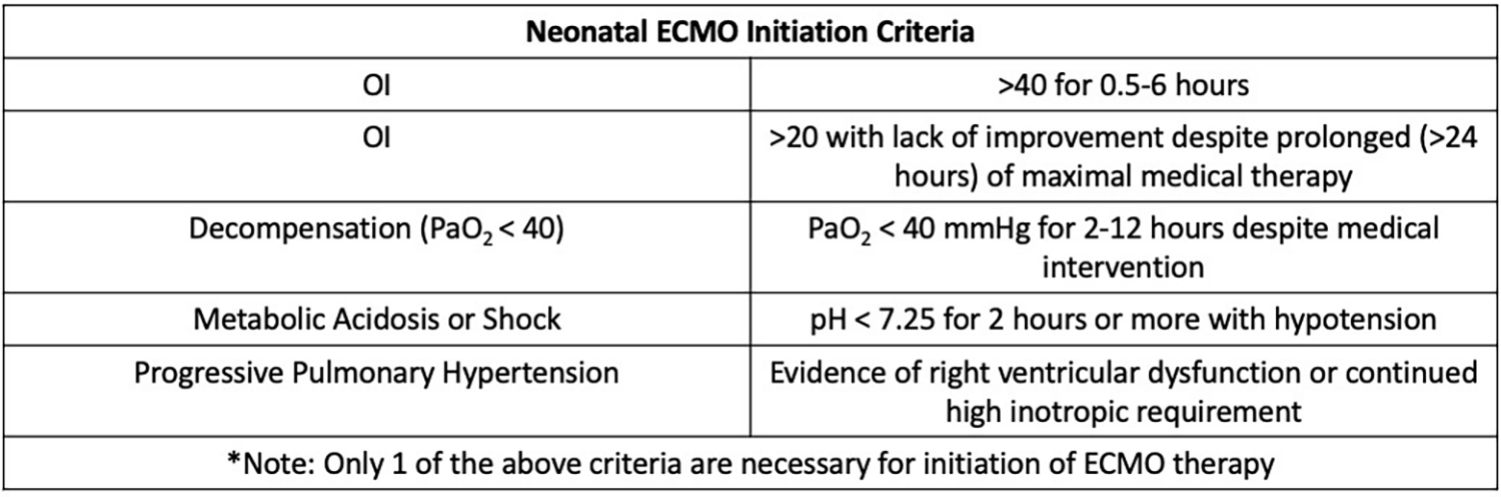
Neonatal ECMO initiation criteria adapted from the ELSO Red book, 6th edition (4).

Extracorporeal membrane oxygenation (ECMO) is a critical life-saving intervention for neonates with respiratory or cardiac failure refractory to conventional treatments. Clinical guidelines for ECMO initiation, such as those established by the Extracorporeal Life Support Organization (ELSO), rely on a combination of physiological parameters to assess disease severity. Among these, the oxygenation index (OI) is the most widely used standard (1–4) (Figure 1). It has demonstrated utility not only in guiding ECMO initiation but also in other interventions, such as initation of nitric oxide therapy (5, 6).

**Figure 2:**
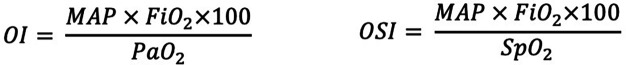
Oxygenation index (OI) and oxygen saturation index (OSI) equations. MAP, mean airway pressure (cmH2O); FiO_2_, fraction of inspired O2 (%); PaO_2_, partial pressure of O_2_ in arterial blood (mmHg); SpO_2_, oxygen saturation via pulse oximetry (%).

The OI is calculated using the formula: OI = (MAP × FiO_2_ × 100)/PaO_2_, where MAP is the mean airway pressure, FiO_2_ is the fraction of inspired oxygen, and PaO_2_ is the arterial oxygen partial pressure. However, this method depends on arterial blood sampling to obtain PaO_2_, which may not always be feasible due to technical or clinical limitations. Additionally, the calculation of the OI typically requires post-ductal PaO_2_ values to accurately reflect pulmonary oxygenation, which are not always obtainable in neonates with severe respiratory compromise (7) (Figure 2).

The oxygen saturation index (OSI) offers a promising non-invasive alternative by substituting pulse oximetry-derived oxygen saturation (SpO_2_) for PaO_2_ (Figure 2). By eliminating the need for arterial blood sampling, OSI is particularly valuable in scenarios where arterial access is difficult or when non-invasive monitoring is preferred (8, 9). Although prior studies have shown a strong correlation between OI and OSI in assessing hypoxemic respiratory failure, there is limited evidence on OSI's reliability in determining ECMO candidacy (10).

This study evaluates the correlation between OI and OSI in neonates requiring ECMO to determine whether OSI can serve as a viable adjunct or alternative for guiding ECMO initiation. Additionally, we examine the thresholds and predictive value of OSI in this context, building on prior research that highlights its potential in neonatal care.

## Methods

This was a retrospective, single-center chart review conducted at Kentucky Children's Hospital, a tertiary care facility specializing in neonatal care. The study included neonates who underwent ECMO between January 2012 and December 2022. A total of 64 neonates requiring ECMO during the study period were identified. Inclusion criteria consisted of neonates admitted for hypoxic respiratory failure and documentation of sufficient data to calculate both the OI and OSI values. Exclusion criteria included incomplete data that precluded calculation of paired OI and OSI values and extreme outlier values, defined as physiologically implausible measurements. Demographic and clinical data, including gestational age, birth weight, and respiratory support parameters, were collected from electronic medical records. Data points for calculating OI and OSI were recorded in 2-hour intervals during the 12 h preceding ECMO initiation. The OI and OSI equations employed are shown in Figure 2.

Values were categorized as OI-OSI pairs, and singleton OI or OSI measurements without a corresponding value were excluded. Average OI and OSI values were calculated in 2 h intervals for 12 h prior to ECMO initiation, and a one-way ANOVA with *post hoc* multiple comparisons of each time point using Bonferroni correction was employed for both OI and OSI, respectively. Correlation between OI and OSI was analyzed using Pearson correlation, which measures the strength and direction of the linear relationship, and Spearman's rho correlation, which assesses the monotonic relationship, accounting for non-linear patterns. Extreme outliers were reviewed and excluded to maintain analytical validity. A *p*-value threshold of 0.05 was used for statistical tests. The study was approved by the Institutional Review Board (IRB) of the University of Kentucky. All data were de-identified to maintain patient confidentiality.

## Results

**Figure 3:**
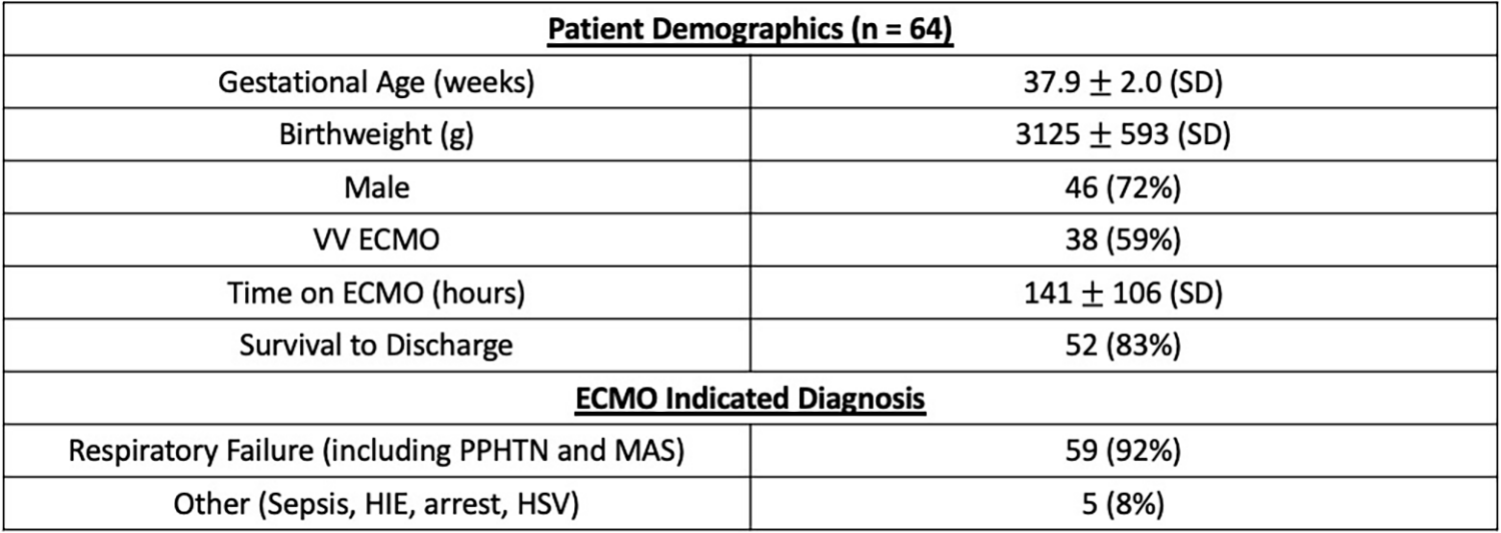
Patient demographic data.

A total of 64 neonates requiring ECMO during the study period met inclusion criteria. Demographic data, including gestational age and birth weight, are summarized in Figure 3. The median gestational age was 37.9 weeks and the median birth weight was 3,125 g. Most neonates were initiated on ECMO due to severe hypoxic respiratory failure.

**Figure 4:**
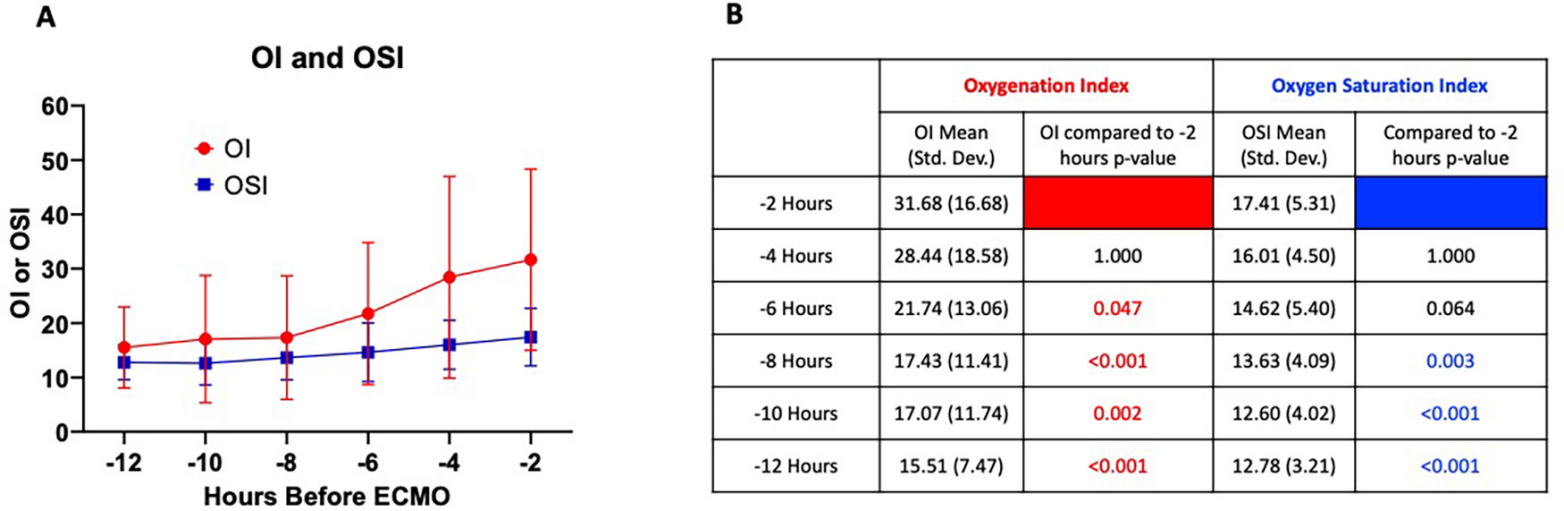
**(A)** Mean OI and OSI values in 2 h increments for 12 h preceding ECMO initiation. Error bars = standard deviation. One-way ANOVA for OI and OSI showed *p* < 0.001 for both, respectively. **(B)** Table of *post hoc* multiple comparisons using Bonferroni correction showing comparison of OI and OSI at −2 h to all other time points. Significant *p*-values <0.05 shown in red and blue for OI and OSI, respectively.

Over the 12 h preceding ECMO initiation, 308 OI and 402 OSI measurements were recorded. After excluding singleton and physiologically implausible values, 298 paired OI-OSI measurements were included in the final analysis. Mean OI and OSI values two hours before ECMO initiation were 31.68 (SD = 16.67) and 17.41 (SD = 5.31), respectively. These values are presented in Figure 4A, which depicts mean OI and OSI values across 2-hour intervals. A one-way ANOVA was performed on both OI and OSI over time, showing both significantly increased prior to ECMO initiation. *post hoc* multiple comparisons using Bonferroni correction of OI and OSI 2 h prior to ECMO initiation to all other time points emphasizes this definite trend of OI and OSI increasing significantly in the preceding hours leading to ECMO initiation (Figure 4B).

**Figure 5:**
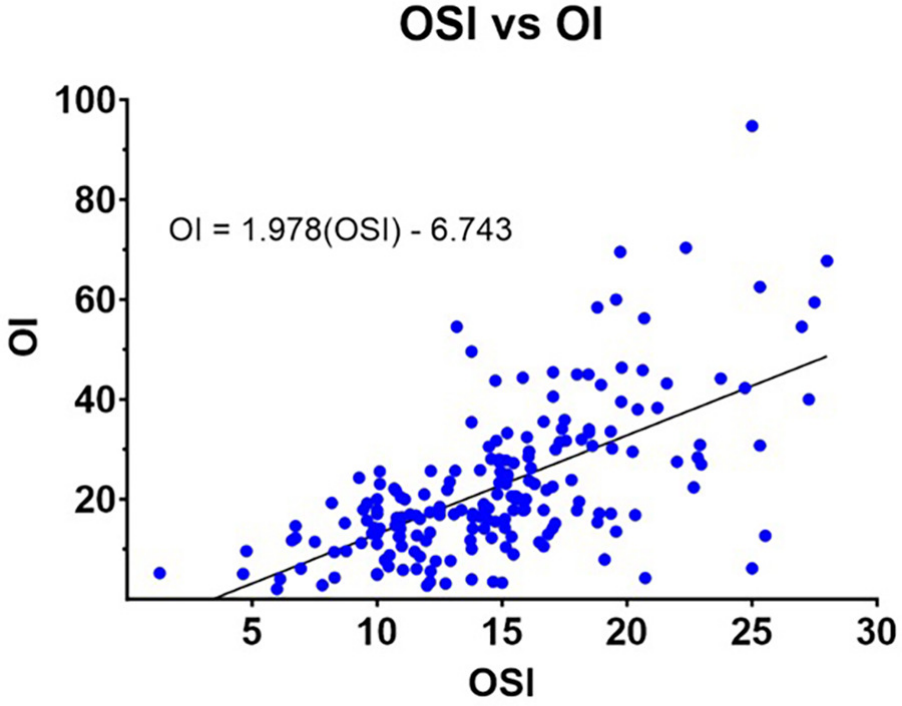
Oi vs. OSI for all data pairs with best fit linear equation.

Correlation analyses demonstrated a moderate positive relationship between OI and OSI. Pearson correlation analysis yielded a coefficient of 0.643 (*p* < 0.001), while Spearman's rho correlation was 0.625 (*p* < 0.001). A linear regression analysis provided the following equation for estimating OI from OSI: OI = 1.978(OSI)—6.743. The scatterplot in Figure 5 illustrates this relationship, with paired OI-OSI measurements and the best-fit regression line.

## Discussion

This study demonstrates a moderate positive correlation between the oxygenation index (OI) and oxygen saturation index (OSI) in neonates who reqeuired ECMO. In our population, an OSI value of 17.41 was identified as a threshold predictive of ECMO initiation, indicating severe respiratory failure that necessitated intervention. These findings highlight the OSI's potential as a non-invasive adjunct to OI in guiding clinical decisions regarding ECMO initiation, and our study shows an OSI of >17.41 for a sustained period of 0.5 to 6 h without improvement despite maximal medical management could be used as criteria for ECMO initiation in situations where calculating an OI wasn't possible.

**Figure 6:**
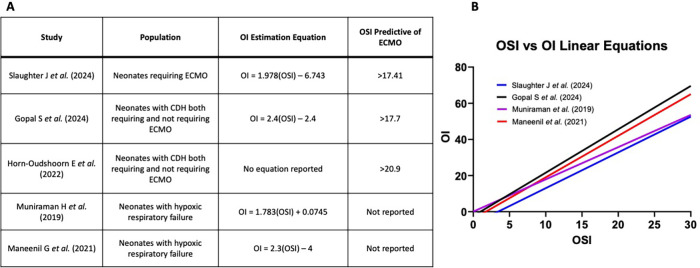
**(A)** Oi estimation equations and OSI values predictive of needing ECMO from various studies. **(B)** Plot of OI Estimation Equations from various studies.

The strong correlation between OI and OSI aligns with prior studies that investigated their relationship in different neonatal populations, including infants with congenital diaphragmatic hernia (3, 10–12). Similar to our results, these studies reported equations for estimating OI from OSI, with comparable thresholds for identifying neonates requiring ECMO (Figure 6). This consistency across studies strengthens the argument for the OSI's clinical utility in neonatal respiratory failure. The derived equation from our study, OI = 1.978(OSI)—6.743, further supports the feasibility of using OSI to approximate OI, particularly in settings where arterial blood sampling is challenging (3, 11, 12).

The identification of an OSI threshold for ECMO initiation offers practical advantages. Arterial blood gas measurements required for OI calculations can be technically difficult to obtain in critically ill neonates, especially in resource-limited settings or situations where frequent arterial sampling is contraindicated. By contrast, OSI relies on pulse oximetry, which is non-invasive, continuous, and widely accessible. As a result, OSI could serve as a valuable alternative or complement to OI, enabling earlier identification of neonates at risk of severe respiratory compromise.

Our findings are particularly relevant in the context of neonatal intensive care units (NICUs) where rapid decision-making is critical. The use of OSI allows for real-time continuous monitoring of respiratory status, providing a continuous parameter that clinicians can track alongside other vital signs, which could lead to more timely interventions for neonates with severe hypoxic respiratory failure. Moreover, the application of OSI could reduce dependency on invasive procedures, minimizing discomfort and risk for vulnerable patients.

Despite its promise, OSI should not be viewed as a replacement for OI but rather as a complementary tool. The OI remains the clinical standard for determining neonatal ECMO candidacy (4). However, in clinical scenarios where arterial blood sampling is challenging, OSI offers a pragmatic alternative that retains significant predictive power. Our study contributes to a growing body of evidence supporting the integration of OSI into routine clinical practice, particularly for neonates with severe hypoxic respiratory failure.

Our results are consistent with prior studies that have examined the correlation between OI and OSI in neonatal populations (3, 10–12). For example, studies involving neonates with congenital diaphragmatic hernia reported similar OSI thresholds for ECMO initiation, further validating the predictive utility of OSI (10). These studies also demonstrated comparable equations for estimating OI from OSI, reinforcing the robustness of our findings. Figure 6 summarizes these comparative data, highlighting the consistency of OSI's performance across diverse patient populations.

However, unlike prior studies that included both ECMO and non-ECMO patients, our analysis focused exclusively on neonates requiring ECMO. While this allowed for precise evaluation of OSI thresholds within this high-risk cohort, it limits the generalizability of the findings to broader neonatal populations. Future research should aim to include neonates with varying severities of respiratory failure to better delineate the role of OSI in the continuum of care.

Several opportunities for further research emerge from this study. Prospective, multicenter studies are needed to validate OSI thresholds across diverse populations and clinical settings. Additionally, exploring the role of OSI in guiding interventions other than ECMO, such as high-frequency ventilation or nitric oxide therapy, could expand its clinical application. Finally, integrating OSI into decision-support systems or predictive models could enhance its utility in real-time clinical practice, potentially leading to more timely intervention for critically ill neonates.

## Limitations

The timing of data collection introduced a challenge in directly aligning OI and OSI values. OI calculation requires arterial blood gas (PaO₂) measurements, which are often obtained intermittently, whereas OSI relies on continuously monitored pulse oximetry (SpO₂). This discrepancy occasionally necessitated the pairing of measurements taken at slightly different times. While the time separation was minimized whenever possible, it may still introduce variability in the correlation analyses. Furthermore, it was also difficult to discern from the medical record whether both blood gasses and pulse oximetry measurements were from pre- or post-ductal sources. A future prospective study is warranted as it would ensure all OI and OSI measurement pairs were taken at the exact same time point and from appropriate post-ductal locations. We believe this type of prospective study would only further strengthen the correlation we observed as well.

Differences in skin pigmentation have been identified as a source of measurement error in pulse oximetry, which may result in inaccurate OSI calculations for certain patient populations included in our data set (13).

Our cohort consisted exclusively of neonates who required ECMO, which limits the generalizability of our findings to broader neonatal populations, including those with severe respiratory failure who recovered without ECMO. For instance, neonates with persistent pulmonary hypertension of the newborn (PPHN) or other critical conditions would not have been represented in this dataset if they avoided ECMO through other interventions. Although we did not include infants with severe hypoxic respiratory failure who did not require ECMO, many of the studies we compared our results to did, and their findings were consistent with ours (Figure 6). Future studies that include neonates with varying disease severities, including those who do not require ECMO, will provide a more comprehensive understanding of OSI thresholds.

Lastly, the study was conducted at a single tertiary care center, which may limit the applicability of the results to other institutions with differing patient populations, clinical practices, or resources. For example, centers with limited access to arterial blood gas analysis or pulse oximetry technology may face unique challenges in implementing OSI as an adjunct to OI.

## Conclusion

At two hours before ECMO cannulation, an OSI value exceeding 17.41 was predictive of the need for ECMO initiation. Our analysis demonstrated a moderate correlation between OI and OSI in the 12 h preceding cannulation, supported by both Pearson and Spearman correlation analyses. Furthermore, we derived an equation to estimate OI from OSI [OI = 1.978(OSI)—6.743.] which closely approximates with other studies' OI estimation equations (Figure 5). These findings highlight the clinical utility of OSI as a non-invasive adjunct to OI, particularly in scenarios where arterial blood sampling is challenging or impractical, and that sustained OSI of >17.41 for 0.5 to 6 h could be used as criteria to initiate ECMO in situations where calculating an OI was not possible. By providing a reliable alternative, OSI can facilitate earlier and more accessible identification of neonates requiring ECMO, complementing the standard use of OI in neonatal intensive care settings.

## Data Availability

The raw data supporting the conclusions of this article will be made available by the authors, without undue reservation.
